# Supplementary material for: Multidimensional factors influencing continuance usage intention of university library self-service systems: An empirical analysis based on an extended TAM-UTAUT
Source: PLoS One. 2026 Mar 30;21(3):e0346014. doi: 10.1371/journal.pone.0346014 (PMC13035133; doi:10.1371/journal.pone.0346014)
Supplement: S1 File — (DOCX) [file pone.0346014.s001.docx]

**Appendix 1: Core Items of the Questionnaire**

| **Latent Variables** | **Items** |
| --- | --- |
| Navigation | The menu structure of the SSS is clear and easy to understand. |
|  | I can quickly find the required functional modules |
|  | The search function of the system is efficient and accurate |
| Terminology | The terminology on the system interface is easy to understand |
|  | The terminology used by the system is consistent with my cognitive habits |
|  | The consistency of terminology helps me reduce misoperations |
| Relevance | The resources recommended by the system are highly relevant to my needs |
|  | The search results can accurately match my search intent |
|  | The personalized recommendations provided by the system meet my academic needs |
| Accessibility | I can use the SSS whenever I need it (e.g., 24/7 availability) |
|  | The system supports multi-terminal access (e.g., computer, mobile phone) |
|  | The system has a fast response speed and low failure rate |
| Retrieval Knowledge | I am familiar with basic literature retrieval methods (e.g., keyword search) |
|  | I can use advanced retrieval skills (e.g., Boolean logic, field limitation) |
|  | I understand the classification system and metadata rules of library resources |
|  | I can quickly screen useful search results |
| Perceived  Usefulness | Using the SSS can improve the efficiency of my resource acquisition |
|  | The system simplifies processes such as borrowing and renewal |
|  | The functions of the system meet my academic research/learning needs |
|  | Using the system can save my time |
|  | The personalized services of the system are very helpful to me |
|  | Overall, the system has high practical value for me |
| Perceived  Ease of Use | I think it is easy to learn to use the system |
|  | The operation process of the system is simple and intuitive |
|  | I can operate the system easily and proficiently |
|  | The system interface is user-friendly and easy to understand |
|  | No professional technical background is required to use the system |
|  | When encountering problems, I can quickly find solutions |
| continuance usage intention | I am willing to continue using the SSS in the future |
|  | I will prefer to use the SSS rather than manual services |
|  | I will recommend the SSS to others |
|  | Even if there are other alternatives, I still tend to use the system |
|  | I expect to use the system more frequently in the future |
|  | Overall, I hold a positive attitude towards continuously using the system |
