# Supplementary material for: Multidimensional factors influencing continuance usage intention of university library self-service systems: An empirical analysis based on an extended TAM-UTAUT
Source: PLoS One. 2026 Mar 30;21(3):e0346014. doi: 10.1371/journal.pone.0346014 (PMC13035133; doi:10.1371/journal.pone.0346014)
Supplement: Attachment — Submitted filename: Response to Reviewers.docx [file pone.0346014.s003.docx]

Dear Reviewer #1,

We sincerely appreciate your valuable feedback and constructive suggestions on our manuscript. These comments are instrumental in enhancing the quality of our work. We have carefully reviewed and analyzed each point raised and have revised the manuscript accordingly. Below, we detail our primary modifications and responses.

**Response to Comment 1: Regarding business needs vs. technology acceptance and the consideration of mandatory usage.**

Your insightful observation is well-received. We fully concur that users’ continuance intention towards Library Self-Service Systems (SSS) is influenced not only by technology acceptance but more fundamentally by their underlying business needs for the service. Furthermore, adjustments in library service models (e.g., reduction of staffed services, extended SSS operating hours) potentially creating a “mandatory usage context” could indeed affect the purity of continuance intention data. These are crucial theoretical perspectives not sufficiently addressed in our initial model development.

**Specific Revisions:**

**（1）Enhanced Theoretical Analysis in Model Development:** A new subsection, “The Interplay of Business Needs and Technology Acceptance,” has been added to the “Research Model Development” section. This section integrates relevant theories from the Information Systems (IS) continuance literature (e.g., the Expectation- Confirmation Model for IT - ECM-IT) concerning the relationship between “expectations” and “needs,” alongside perspectives on “core service quality” from service marketing, to substantiate this analysis.

**（2）Clarification of Limitation in Discussion:** Within the “Research Limitations and Future Directions” section, we explicitly acknowledge the insufficient consideration of the “mandatory usage context.” We state that data collection occurred within operational library environments where users might utilize SSS due to objective constraints (e.g., limited access to staffed services), potentially introducing a non-volitional component into the measured “continuance intention.”

**Response to Comment 2: Regarding the scope definition of University Library SSS and description of SSS status at the three universities.**

Your point is critically important. Clearly defining the research object is fundamental to ensuring methodological rigor and result interpretability. Our initial description of the specific SSS types studied and the operational realities of SSS at the surveyed universities was indeed insufficient.

**Specific Revisions:**

**Definition of SSS Scope in Introduction/Literature Review:** We have incorporated a clear definition of “University Library Self-Service Systems (SSS)” within the “Introduction” section. This definition specifies the types of SSS encompassed in this study, briefly described as: **Self-Checkout Terminals:** For self-service borrowing, returning, and renewing of physical library materials. **Online Public Access Catalog (OPAC) Systems:** For searching and retrieving information on both physical and digital library holdings.**Self-Service Copying/ Printing/Scanning Terminals:** Providing self-service document copying, printing, and scanning. **Electronic Resource Remote Access Platforms:** Platforms (accessed via campus network or off-campus authentication) for databases, e-journals, e-books, and other digital resources. **Description of SSS Context in Data Collection Section:** A new paragraph has been added to the “Data Collection” subsection, briefly outlining the current SSS deployment and service profiles at Qinghai University, Qinghai Normal University, and Qinghai Nationalities University. This includes: The primary SSS types deployed at each library (mapped to the defined scope above). Approximate number of devices (where feasible/relevant). Service coverage (e.g., campus-wide availability, 24-hour access for specific services). Key measures implemented by the libraries to promote SSS adoption in recent years (e.g., reducing staffed service points, enhancing user training on SSS). This also implicitly addresses the “mandatory usage” context. Mention of approximate SSS usage rates at each institution, if obtainable, to provide fuller context.

**Response to Comment 3: Regarding inclusion of recent relevant Library and Information Science (LIS) literature.**

Thank you for recommending two highly relevant and excellent studies. We acknowledge that our literature review could have provided a more comprehensive synthesis of recent LIS research applying TAM/UTAUT and their extensions to study the continuance use of library resources or services.

**Specific Revision:**

**Enhanced Literature Review:** Within the “Literature Review” section, specifically under “Research on Library Self-Service Systems” or a dedicated subsection on “Application of Technology Acceptance Models in the Library Domain,” we have incorporated a review and discussion of the two recommended studies. We compare and contrast their findings and frameworks with our own theoretical model and research questions, highlighting similarities, differences, and the potential contribution of our study to this evolving literature stream. Correct citation formatting is ensured, and the references are added to the list.

**Response to Comment 4: Regarding the confusion between "Ethnicity" and "Nationality".**

We sincerely apologize for this significant oversight during manuscript preparation and proofreading, which led to conceptual confusion. We fully understand that “Ethnicity” (Minzu) and “Nationality” are distinct concepts. All participants in this study are Chinese citizens, and our focus is on potential differences arising from diverse “Ethnicity” backgrounds within China, such as cultural adaptation or usage patterns.

**Specific Revisions:**

**Comprehensive Textual Correction:** We have meticulously reviewed the entire manuscript. All instances where “Ethnicity” was erroneously labeled or described as “Nationality” in tables (e.g., Table 1 “Respondents’ characteristics,” Table 3, Table 4, Table 7) and textual descriptions have been corrected to “Ethnicity (Minzu)”.

**Conceptual Clarification:** Where “Ethnicity” is first introduced as a moderating variable (e.g., in the “Research Hypotheses - Moderating Variables” or “Results - Moderating Effect Analysis” sections), we have added a brief clarification stating that it refers to “Chinese Ethnic Groups (Minzu)” to prevent any ambiguity.

**Enhanced Proofreading:** We will implement stricter checks for terminological and translational accuracy in subsequent revisions to prevent recurrence.

Once again, we express our deepest gratitude for your meticulous review and highly constructive feedback. These revisions will undoubtedly enhance the scholarly rigor and theoretical depth of our study. We will submit the revised manuscript promptly.

Sincerely,

The Author Team

Dear Reviewer #2,

We sincerely appreciate your valuable feedback and detailed suggestions on our manuscript. Your comments are highly constructive and provide essential guidance for enhancing the quality and rigor of our paper. We have carefully studied and analyzed each point raised and have revised the manuscript accordingly. Below are our detailed responses to your specific concerns and the corresponding amendments implemented.

**Major Issues: Responses and Revisions**

**1.Regarding the proportion of librarians/library staff in the sample, sensitivity analysis of results, inclusion of age as a variable, and statistical quantification of demographic differences:**

We thank you for highlighting this critical issue. In the original manuscript, we indeed failed to clearly specify the proportion of librarians and library staff within the sample and its potential impact on the model results. We also neglected to adequately incorporate age as a key variable into the model and discuss its implications. Furthermore, the analysis of demographic differences lacked specific statistical quantification.

**(1) Proportion of Librarians/Library Staff:** Among the 365 valid respondents, librarians and library staff totaled 28 individuals, constituting 7.67% of the total sample.

**(2) Sensitivity Analysis of Results:** We acknowledge that this group, due to their professional background, may possess a deeper understanding of library self-service systems and different usage motivations, potentially influencing the model estimates.

**(3) Age Variable:** Age is indeed a significant factor influencing technology acceptance and usage behavior, particularly concerning the distinction between "technology hesitancy (older adults)" and "technology comfort (Gen Z)".

**(4) Statistical Quantification of Demographic Differences:** Your observation regarding the lack of statistical significance tests and quantitative results when discussing gender and ethnicity differences is valid; this omission did weaken the persuasiveness of our conclusions.

**Revisions Implemented: To address this important concern, we have undertaken the following steps:**

**(1) Supplemented Sample Structure Information:** We have added a new row labeled "Occupation/Status" in Table 1 (Respondents’ characteristics) within the "Data Collection" section, detailing the number and percentage of library staff and other respondents.

**(2) Conducted Sensitivity Analysis: We have performed supplementary analyses:**

Added "Is a librarian/library staff member" as a moderator variable, named "Occupation," within the research model framework (Section 2.3).

Incorporated the moderating effects of Occupation, Gender, Ethnicity, and Usage Frequency into the variable model. We re-ran the moderation analysis using PLS-SEM with interaction terms to test their effects on the core paths (e.g., Perceived Ease of Use → Continuance Intention, Perceived Usefulness → Continuance Intention, Retrieval Knowledge → Continuance Intention).

Integrated these new findings into the "Moderating Effect Analysis" section and discussed the mechanisms of influence related to Occupation, Gender, Ethnicity, and Usage Frequency within the "Discussion and Conclusions."

**(3) Acknowledged Age Variable Limitation:** As the questionnaire did not include age as a demographic variable, we have explicitly stated this limitation within the "Research Limitations and Future Work" section.

**2.Regarding Inaccessibility of the Questionnaire and Response Data:**

We sincerely apologize for the inconvenience this caused during your review. We understand that transparency regarding data and instruments is paramount for assessing the technical quality of the research and the reproducibility of the results.

(1) We are preparing the survey questionnaire (to be translated into English) and the anonymized response dataset (containing no personally identifiable information). We plan to upload these materials to a publicly accessible academic data repository, such as Figshare, Dryad, or the Open Science Framework (OSF). Once uploaded, we will provide a clear, permanent link (DOI) in the "Data Availability Statement" section of the paper, facilitating access for you and other readers. Additionally, we will include a temporary access link or instructions for retrieval within our response letter accompanying the revised manuscript.

**Minor Issues: Responses and Revisions**

**1.Regarding Broadening SEM Literature Understanding and Citation, Including Library Domain SEM Analyses:**

We appreciate your suggestion. We recognize that our current citations for SEM literature are concentrated on Hair et al.'s authoritative work. Broadening the sources will enhance the comprehensiveness of our study and understanding of SEM applications in specific domains like library science.

**Revision Implemented:** We have systematically searched for and incorporated citations of relevant studies employing SEM within the library science field (e.g., exploring library service quality, digital resource usage behavior, user satisfaction). We have also cited influential works and recent advancements by other prominent SEM scholars (e.g., Anderson, J.C., Gerbing, D.W., Bollen, K.A., Byrne, B.M.) to enrich the theoretical foundation and methodological perspective.

**2.Regarding the Overly Long and Specific Title, Request to Rewrite in Understandable Language Without Abbreviations:**

Your point is well-taken. The original title did contain excessive jargon and abbreviations, potentially hindering comprehension for non-specialist readers and limiting the paper's dissemination.

**Revision Implemented:** We have rephrased the title to be concise, clear, and accessible, avoiding abbreviations while highlighting the core contribution. For example: "Multidimensional Factors Influencing Continuance Usage of University Library Self-Service Systems: An Empirical Analysis Based on an Extended Technology Acceptance Model" or "Factors Influencing Users' Continuance Intention Towards University Library Self-Service Systems: An Integrated Model and Multi-Group Analysis" (We will finalize the title that most accurately reflects the study content).

**3.Regarding Lack of Contextualization of University Library Discussion within the Chinese Setting:**

This was a significant oversight. University libraries in China operate within unique contexts regarding smart campus initiatives, resource development strategies, and user needs, influenced by specific policies. Placing the study within this context enhances its situational validity and practical significance.

**Revisions Implemented:**

We have added a dedicated paragraph in the "Introduction" outlining the unique background of Chinese university library development, covering: National initiatives promoting "Smart Education" and "Education Informatization 2.0" driving library digital transformation. Challenges posed by university enrollment expansion and growing student/faculty numbers on library service capacity. Characteristics of collection development in Chinese university libraries (especially Chinese resources and specialized databases). The current state and common challenges (e.g., regional disparities, varying user digital literacy) in adopting self-service technologies within Chinese university libraries. We have revised the phrase "exponential growth of collection scale" to a more precise statement: "With the development of higher education and the exponential growth of information resources, both the collection resources and user demands in Chinese university libraries have shown rapid growth trends." We will also consider citing relevant Chinese university library statistical reports for support.

**4.Regarding Using Table Numbers in Text for Reader Navigation:**

Thank you for this reminder. Explicitly referencing table numbers in the text effectively guides readers and improves readability.

Revision Implemented: We have meticulously reviewed the entire manuscript to ensure that every table is explicitly referenced by its number upon first mention or discussion (e.g., "As shown in Table 1...", "This result can be seen from the path analysis in Table 2...").

**5.Regarding Spelling Errors and Other Textual Issues:**

**Response:** We sincerely apologize for these oversights, which reflect shortcomings in our proofreading process. Revisions Implemented: We have conducted a thorough proofread and edit to correct all spelling, grammatical, and phrasing errors:

1）Line 27: "continuously" changed to "continuous".

2）Line 46: "applicability" adjusted contextually (e.g., "While TAM and UTAUT models have broad applicability, their sufficient application in dynamic academic library environments requires further exploration" or "their explanatory power in specific contexts may not be fully tapped").

3）Lines 103+ (PU and PEOU): Adjusted phrasing to use full terms followed by abbreviations in parentheses upon first mention (e.g., "Perceived Usefulness (PU)", "Perceived Ease of Use (PEOU)"). Abbreviations are used consistently thereafter.

4）Line 121 (TAM Limitations): Strengthened the logical link between the transition sentence (Line 119) and the limitations description (e.g., "Despite the success of the TAM in explaining technology acceptance behavior, limitations remain in explaining continuance usage within complex service environments. Specifically, the TAM does not sufficiently account for social influence, individual user differences, and the formation of long-term usage habits...").

5）Lines 157-158 (Model Mapping): Created a table clearly listing the constructs in our model, their original theoretical sources (TAM/UTAUT/New Variables), and example measurement items to aid reader comprehension of the framework.

6）Line 210 (H4): Explicitly discussed the potential influence of the "library staff/librarian" group on the "Retrieval Knowledge" construct. Added sample distribution details for different occupations/statuses in "Data Collection" and analyzed potential bias and its impact on H4 results in "Discussion" or "Limitations".

7）Line 218: "positive" changed to "positively".

8）Lines 310 & 318 (Heading Size): Adjusted heading levels to be clear subheadings (e.g., "3.1 Reliability Assessment", "3.2 Validity Assessment") to distinguish analysis steps clearly and link them to preceding tables.

9）Line 397 (Table Title): "moderate" changed to "moderation" (e.g., "Table 7: Moderation Analysis Results").

10）Line 421 (R² Values): We agree. We now report R² values avoiding undue emphasis on minute differences beyond the second decimal place unless statistically and substantively significant. We focus on reporting major, meaningful changes. Statistical indicators (β, p, f²) are provided for all key findings.

11）Line 437: "combines" changed to "uses".

Once again, we extend our deepest gratitude for the considerable time and effort you dedicated to meticulously reviewing our manuscript and providing such constructive feedback. We have diligently addressed all the points raised above to improve the paper's quality. The revised manuscript will be submitted for your review promptly.

Sincerely,

The Authors.

Dear Reviewer #3,

Thank you very much for your valuable feedback and positive assessment of our research paper. Your suggestions provide crucial guidance for enhancing the paper’s structural clarity and academic rigor. We have carefully addressed your comments with the following revisions:

**1.Regarding your suggestion to "give theoretical implications and managerial implications their own separate sections":**

**Modification:** Within the "Discussion and Conclusions" section, we have restructured the original integrated discussion. We have explicitly added two distinct subsections/paragraphs: "Theoretical Implications" and "Managerial Implications".

Theoretical Implications: This new subsection consolidates our discussion of the study's contributions to the literature and novel theoretical insights. It focuses on extending Technology Acceptance theories (specifically TAM and UTAUT), integrating multi-dimensional influencing factors, and the discovery of moderating effects. For instance, it emphasizes the theoretical significance of introducing variables like information retrieval knowledge and accessibility into the model, and the value of validating and enriching technology acceptance theory within the specific context of academic libraries.

Managerial Implications: This dedicated subsection provides concrete, actionable recommendations for practitioners – specifically library administrators, system developers, and service providers. It addresses practical aspects such as system optimization, user training, service promotion, and differentiated management strategies based on our findings. Examples include improving system navigation and terminology design, enhancing users' information retrieval knowledge, and addressing the needs of diverse user groups (e.g., different genders, ethnicities, usage frequencies).

**2.Regarding your suggestion to "write out full forms of specialized terms upon first use in the Introduction, e.g., SSS":**

Modification: We have thoroughly examined the "Introduction" section to ensure all specialized terms are written out in full upon their first occurrence, accompanied by abbreviations and necessary explanations. Specifically:

The first mention of "Self-services system" now explicitly states: "Self-Service System (SSS)".

Similarly, other key terms such as "Self-Service Technology (SST)", "Technology Acceptance Model (TAM)", "Unified Theory of Acceptance and Use of Technology (UTAUT)", and "Partial Least Squares Structural Equation Modeling (PLS-SEM)" are presented with their full forms and abbreviations upon their initial introduction in the Introduction and respective chapters.

We believe these revisions significantly improve the paper's structure, logical flow, and terminological precision, better meeting the standards for academic publication. We sincerely appreciate your thoughtful guidance.

Best regards,

The Authors
