# Supplementary material for: Multidimensional factors influencing continuance usage intention of university library self-service systems: An empirical analysis based on an extended TAM-UTAUT
Source: PLoS One. 2026 Mar 30;21(3):e0346014. doi: 10.1371/journal.pone.0346014 (PMC13035133; doi:10.1371/journal.pone.0346014)
Supplement: Attachment — Submitted filename: Response_to_Reviewers_auresp_2.docx [file pone.0346014.s004.docx]

Dear Mr. Oriel Jerome Delas Alas Vida and Mr. Adrian Cyrus Luczon,

Greetings!

Thank you for your email notification and patient guidance. I have received the revision requirements from the PLOS ONE editorial office regarding my manuscript titled “Multidimensional Factors Influencing Continuance Usage Intention of University Library Self-Service Systems: An Empirical Analysis Based on an Extended TAM-UTAUT” (Manuscript ID: PONE-D-25-28505R2). My response is as follows:

Regarding the requirement to “remove figures from the manuscript file and retain only individual TIFF/EPS format image files”, I have completed the necessary adjustments to ensure all figures are submitted separately in the standardized format, strictly adhering to the journal’s submission guidelines.

Thank you for your support and assistance with this submission. Please feel free to inform me if there are any additional matters requiring attention.

Sincerely,

Kai Cao

Qinghai University

Email: [caokai19910101@gmail.com](mailto:caokai19910101@gmail.com)
