# Supplementary material for: Multidimensional factors influencing continuance usage intention of university library self-service systems: An empirical analysis based on an extended TAM-UTAUT
Source: PLoS One. 2026 Mar 30;21(3):e0346014. doi: 10.1371/journal.pone.0346014 (PMC13035133; doi:10.1371/journal.pone.0346014)
Supplement: Attachment — Submitted filename: Response_to_Reviewers_auresp_3.docx [file pone.0346014.s005.docx]

Dear Reviewer 2:

We sincerely appreciate your recognition of this paper and your thorough review! The issues you raised regarding the Figshare dataset are precise and critical, providing significant guidance for improving the rigor of the methodology in this study. We highly value your comments and have immediately carried out data organization. The specific revisions are detailed below for your further review.

Regarding your first comment: The number of questionnaire observation items exceeds the number of respondents.Upon checking, we confirm that this issue resulted from an operational error during data upload. We have completed data cleaning and removed all redundant observation items, ensuring that the number of questionnaire observation items is fully consistent with the number of respondents. The cleaned dataset has been re‑uploaded to the Figshare platform and is available for your verification at any time.

Regarding your second comment: No codebook/data dictionary for reproducing the analysis.We have urgently prepared and finalized a codebook and data dictionary, which elaborate on the dataset structure, variable definitions, coding rules, missing value handling, and detailed procedures for all statistical analyses in this paper. This document has been uploaded to Figshare as supplementary material together with the cleaned dataset to ensure that you and other researchers can successfully reproduce the analyses reported in this study.

Regarding your third comment: Inconsistency between perceived usefulness (PU) items in the appendix and those in the dataset (6 items in the appendix vs. only PU1–PU4 in the dataset).This inconsistency was caused by an oversight during the drafting of the appendix—two PU items used only in the pre‑test but not in the formal survey were mistakenly included. The formal survey adopted exactly four items (PU1–PU4), which are fully consistent with those in the dataset. We have revised the appendix by removing the two redundant pre‑test items, ensuring strict consistency between the items listed in the appendix and those in the dataset. We have also documented this adjustment in the revision note to avoid subsequent misunderstanding.

We sincerely thank you again for your patient guidance and valuable comments! Following your suggestions, we have completed all data organization, appendix corrections, and supplementary file uploads to ensure the rigor of the methodology. The improvement of this paper would not have been possible without your assistance. Should you have any further questions or additional recommendations, we will promptly respond and revise accordingly. We sincerely hope you will be satisfied with the revisions and support the publication of this paper.

Yours sincerely,

Authors

March 12, 2026
